# Outcomes and Costs of the Transition From a Paper-Based Immunization System to a Digital Immunization System in Vietnam: Mixed Methods Study

**DOI:** 10.2196/45070

**Published:** 2024-03-18

**Authors:** Thi Thanh Huyen Dang, Emily Carnahan, Linh Nguyen, Mercy Mvundura, Sang Dao, Thi Hong Duong, Trung Nguyen, Doan Nguyen, Tu Nguyen, Laurie Werner, Tove K Ryman, Nga Nguyen

**Affiliations:** 1 National Expanded Program on Immunization National Institute of Hygiene and Epidemiology Hanoi Vietnam; 2 PATH Seattle, WA United States; 3 PATH Hanoi Vietnam; 4 Bill & Melinda Gates Foundation Seattle, WA United States

**Keywords:** eHealth, digital health, immunization information system, electronic immunization registry, immunization, data quality, data use, costing

## Abstract

**Background:**

The electronic National Immunization Information System (NIIS) was introduced nationwide in Vietnam in 2017. Health workers were expected to use the NIIS alongside the legacy paper-based system. Starting in 2018, Hanoi and Son La provinces transitioned to paperless reporting. Interventions to support this transition included data guidelines and training, internet-based data review meetings, and additional supportive supervision visits.

**Objective:**

This study aims to assess (1) changes in NIIS data quality and use, (2) changes in immunization program outcomes, and (3) the economic costs of using the NIIS versus the traditional paper system.

**Methods:**

This mixed methods study took place in Hanoi and Son La provinces. It aimed to analyses pre- and postintervention data from various sources including the NIIS; household and health facility surveys; and interviews to measure NIIS data quality, data use, and immunization program outcomes. Financial data were collected at the national, provincial, district, and health facility levels through record review and interviews. An activity-based costing approach was conducted from a health system perspective.

**Results:**

NIIS data timeliness significantly improved from pre- to postintervention in both provinces. For example, the mean number of days from birth date to NIIS registration before and after intervention dropped from 18.6 (SD 65.5) to 5.7 (SD 31.4) days in Hanoi (*P*<.001) and from 36.1 (SD 94.2) to 11.7 (40.1) days in Son La (*P*<.001). Data from Son La showed that the completeness and accuracy improved, while Hanoi exhibited mixed results, possibly influenced by the COVID-19 pandemic. Data use improved; at postintervention, 100% (667/667) of facilities in both provinces used NIIS data for activities beyond monthly reporting compared with 34.8% (202/580) in Hanoi and 29.4% (55/187) in Son La at preintervention. Across nearly all antigens, the percentage of children who received the vaccine on time was higher in the postintervention cohort compared with the preintervention cohort. Up-front costs associated with developing and deploying the NIIS were estimated at US $0.48 per child in the study provinces. The commune health center level showed cost savings from changing from the paper system to the NIIS, mainly driven by human resource time savings. At the administrative level, incremental costs resulted from changing from the paper system to the NIIS, as some costs increased, such as labor costs for supportive supervision and additional capital costs for equipment associated with the NIIS.

**Conclusions:**

The Hanoi and Son La provinces successfully transitioned to paperless reporting while maintaining or improving NIIS data quality and data use. However, improvements in data quality were not associated with improvements in the immunization program outcomes in both provinces. The COVID-19 pandemic likely had a negative influence on immunization program outcomes, particularly in Hanoi. These improvements entail up-front financial costs.

## Introduction

### Background

Since 2017, the National Immunization Information System (NIIS) in Vietnam has been used nationwide by immunization facilities from the national, provincial, district, and commune levels to capture, store, and access immunization data [1]. The NIIS is a digital system that includes an immunization registry comprising individual-level, longitudinal information on vaccine doses administered and a logistics management system for vaccines and related supplies. As of June 2022, the NIIS has recorded data from approximately 31 million clients and has been used across 15,000 immunization facilities.

Although there is a growing body of evidence on the outcomes associated with digital systems to support vaccine service delivery in low- and middle-income countries [2-9], there are still many questions regarding how they affect data quality, use, and vaccination outcomes in practice. A challenge that has emerged in multiple country contexts is the parallel or dual reporting required when digital systems are introduced and health workers are expected to continue to use the legacy paper-based forms in addition to the new digital system. For example, Tanzanian facilities that had transitioned entirely to using an electronic immunization registry had higher odds of system use compared with those maintaining parallel electronic and paper-based systems [10].

Moreover, there is limited evidence on the costs of development and implementation of the system, recurrent costs of the system, and cost implications of eliminating a parallel paper system at the service delivery level [11,12]. The Vietnamese experience of introducing and scaling digital tools for immunization presents an opportunity to help fill these evidence gaps.

### History of the NIIS

Before the introduction of the NIIS, a paper-based immunization registry and vaccine stock management system were used. Health workers captured data on paper forms, which were compiled in a monthly report. The paper-based system created a significant workload for health workers, and the immunization data were often delayed or incomplete, limiting the availability of reliable data at the higher levels of the health system [13].

From 2009 to 2012, the National Expanded Program on Immunization (NEPI), in collaboration with PATH and the World Health Organization, developed and piloted a logistics management tracking system for vaccines and related supplies (VaxTrak) and an immunization registry (ImmReg) at the commune and district levels. After the pilot phase, ImmReg expanded to all districts in the pilot province, and VaxTrak was scaled nationwide. An evaluation of ImmReg in 2015 showed that the system was highly accepted by health workers and improved vaccine coverage and timeliness [6]. In 2014 and 2015, NEPI and PATH integrated ImmReg and VaxTrak into a single comprehensive software system. From 2016 to 2018, the Vietnam General Department of Preventive Medicine and PATH developed the NIIS based on the pilot software and scaled it nationwide [1].

### Transition to a Paperless System

Beginning in 2018, the government of Vietnam collaborated with PATH to provide technical support to strengthen NIIS implementation and transition to a paperless system in 2 provinces: Hanoi and Son La. This work was funded by the Bill & Melinda Gates Foundation and was implemented from 2018 to 2022.

We hypothesized that transitioning to a paperless system would improve data quality and data use in the intervention areas. We hypothesized that if data quality and data use improved, these changes could lead to improvements in immunization program outcomes. The objective of this study was to examine the short-term outcomes and costs associated with the NIIS and the transition to paperless reporting, focusing on three main categories:

1. What are the changes in data quality and data use because to the paperless transition interventions?
2. What are the changes in immunization program outcomes (timeliness, dropout rates, and coverage) because of the paperless transition interventions?
3. What are the incremental financial costs associated with developing, deploying, and maintaining the NIIS, including the economic cost implications of transitioning to paperless reporting?

## Methods

### Overview

This mixed methods study aimed to evaluate the short-term outcomes and costs associated with the transition to a paperless system. Mixed methods were used to quantify the observed changes in outcomes and costs and to qualitatively describe why and how changes occurred. Pre-post analyses were conducted to understand the short-term changes in data quality, data use, and immunization program outcomes. Financial data were extracted from the project and partners’ records, and interviews were conducted at various levels of the health system to inform the cost analysis. This study was conducted from July 2019 to November 2021.

### Setting

This study was conducted in 2 intervention provinces, Hanoi and Son La. These provinces were selected for the transition to a paperless system because they have a variety of geographic, demographic, and health system characteristics that may influence digital readiness. Hanoi, the capital city, is primarily urban with a high population density, high immigration rate, good infrastructure, and many private-sector and fee-based facilities. In contrast, Son La is a mountainous border province primarily composed of rural districts and has low population density, limited resources, and fee-based facilities.

Each province provides immunizations in public district health centers, hospitals, and commune health centers (CHCs) as well as private fee-based immunization facilities (FIFs). Primary data collection for this study also occurred at the national level, for example, to capture financial costs from the project and partners related to the development of the NIIS.

The transition to a paperless system and overall immunization activities in Vietnam were impacted by the COVID-19 pandemic, starting in 2020. The government mandated social distancing lockdowns multiple times in each province, which meant that individuals were not allowed to leave their home without special authorization, and nonessential businesses were closed. Immunization services were disrupted and, in some cases, unavailable, as health care workers were occupied with the COVID-19 response.

Multimedia Appendix 1 includes an overview of the characteristics of the study provinces (Table S1 in Multimedia Appendix 1) and dates when COVID-19 social distancing was applied in each province (Table S2 in Multimedia Appendix 1).

### Interventions

A technical working group composed of the Ministry of Health, NEPI, PATH, and Viettel (the NIIS developer) oversees the implementation of the NIIS. A readiness assessment was conducted from June to July 2019 to provide the technical working group with information about the progress, needs, and challenges of transitioning to a fully paperless immunization system [14]. NEPI and PATH designed interventions (summarized in Textbox 1) to support the transition to paperless reporting based on the readiness assessment results. Interventions included detailed implementation guidelines and standard operating procedures for the transition to paperless reporting, internet-based data review meetings, additional supportive supervision visits, and Zalo (Vietnam’s popular social media and chat app) groups for end users to exchange knowledge and experiences in NIIS use.

**Key interventions implemented as part of the transition to paperless reporting.**

**Key interventions**

- *Guidelines and training on the shift to paperless reporting*: implementation guidelines and standard operating procedures for the transition to paperless were implemented through a training of trainers and cascaded training approach for health workers.
- *Data quality and data use guidelines and training* for health workers at the province, district, and commune health center levels.
- *Internet-based data review meetings* at the district level where all communes share progress on paperless transition. Challenges identified through these meetings were used to prioritize areas for support. Initially, these meetings were held monthly but later shifted to quarterly.
- *Additional supportive supervision visits* from the government and PATH at district and commune facilities to support data quality, data use, and the overall transition to paperless. During the COVID-19 pandemic, these shifted to internet-based supportive supervision visits. Internet-based supportive supervision guidelines and trainings were developed for districts and provinces.
- *Zalo groups for end users* (at least 1 National Immunization Information System [NIIS] user per facility) in each district to exchange knowledge and experiences in NIIS use. Zalo is a popular social media and chat app in Vietnam.

Facilities began transitioning to paperless reporting in November 2019 in Son La and in January 2020 in Hanoi. All facilities in both provinces have retired from the paper-based immunization management logbook and have completely transitioned to paperless reporting using the NIIS as of January 2020. Although all sites are officially reporting using the NIIS, some paper-based systems are still used to comply with various inspections, payment procedures, or requirements from other ministries.

In addition to the key interventions (Textbox 1), an e-learning system and e-immunization cards were piloted at a smaller scale. The e-learning system was developed and piloted in 6 districts (in the 2 provinces) to train managers at the national, regional, provincial, and district levels and facility health workers (CHCs, FIFs, and hospitals) on using the NIIS. The e-immunization card, a mobile phone app that allows parents or clients to access their individual demographic information and vaccination data, was also developed and launched in the 2 provinces.

### Study Design and Data Collection

#### Overview

Various methods were used to collect data related to each of the study aims (data quality and use, immunization performance, and costing) in the 2 provinces. This section describes the study design, data sources, and data collection approach for each study objective. Table S3 in Multimedia Appendix 1 summarizes the data collection methods across the 3 study aims.

#### Data Quality and Use

A pre- or postintervention study design was used to assess changes in data quality and use. Data collection included self-administered facility assessments, household surveys, and facility surveys conducted at a sample of CHCs, FIFs, and hospitals; details of the methodology have been published elsewhere [14]. The same methods were used for pre- and postintervention data collection, with the addition of in-depth interviews with Expanded Program on Immunization (EPI) officers at the district and commune levels at postintervention.

The self-administered facility assessment was sent via email to all immunization facilities to collect basic information regarding infrastructure, capacity, and NIIS data use.

Facility surveys were conducted on a purposively selected sample of districts, communes, hospitals, and FIFs in each province. Purposive sampling was performed in consultation with NEPI and provincial Centers for Disease Control and Prevention (CDC) to select a mix of facility types (fee based, private, and public), geographies (urban, semiurban, rural, and mountainous), and experiences with NIIS. The study was designed to survey the same facilities at pre- and postintervention. At preintervention, 8 FIFs and 7 hospitals were included; at postintervention, 5 FIFs and 7 hospitals were included (the change was owing to 3 FIFs that had closed by the time of the postintervention survey). In each facility, 20 clients in the paper logbook were randomly selected. The facility surveys captured structured information about the facility, the use of the NIIS, and demographic and immunization information for the 20 sampled clients.

A household survey was conducted in a sample of households with children aged <2 years to capture demographic and immunization information about the children from their home-based immunization cards. The household survey was conducted in the same purposively selected communes as the facility surveys. Within each commune, villages or living quarters were selected for convenience, and all households with children in the defined age range were included.

The NEPI and PATH staff trained data collectors who used KoboToolbox platform for data entry for all data collection forms. More details on the sampling approach and a full list of facilities included in the different evaluations conducted as part of this study are included in Table S4 in Multimedia Appendix 1.

Structured interviews were conducted to understand the factors related to data quality and data use. CDC staff led the interviews with immunization managers at the district level and health workers at the commune level. Interviews involving NEPI and CDC personnel were performed by the PATH staff. Interviews were conducted over the phone and in person at the interviewees’ workplaces, lasting around 35 minutes. The interviews were recorded with consent and transcribed.

#### Immunization Program Outcomes

A pre- or postintervention study design was used to assess changes in immunization program outcomes in the 2 provinces using NIIS data. NIIS data were exported for a pre- and postintervention cohort of children in each province. Due to the differing commencement dates of paperless reporting in 2 project provinces was different, Son La began in November 2019, while Hanoi followed in Jan 2020. As a result, the preintervention cohort group comprises children born between July 1 and September 30, 2018, while the postintervention group comprises those born between July 1 and September 30, 2020. Each child’s immunization information was analyzed for their first 12 months of life.

#### Costing

The costing study used a mixed methods approach including primary data collection using a microcosting approach and secondary data collection from financial record reviews. The costs of all activities were estimated from the perspectives of the implementing organizations (Hanoi and Son La provinces for NIIS systems use and Viettel, PATH, and NEPI) and hence take the health system perspective. No client costs were included.

We captured the costs for the different activities of implementing the NIIS from the software design and development activities to the deployment, accounting for the costs of the different partners engaged in this process. For the partner leading the software design, development, and deployment, we include the costs of the infrastructure, server, bandwidth, technical support, help desk, training, and maintenance and operations of the system. For the NEPI and the subnational levels, the costs included those for development of training materials, conducting staff training, data entry, meetings, and internet setup, at facilities where it was needed.

Data on the incremental financial costs for designing, developing, and deploying the NIIS software were obtained through the NEPI and partner organization expenditure records review. We also obtained information from each EPI administration and health facility in the sample on the expenditures for NIIS-related training and meetings and other deployment costs, including costs for data back entry and internet or phone setup at facilities.

Data on the recurrent financial costs of the NIIS were obtained via interviews with the head of each facility or person in charge of the facility finances in each study facility in the 2 provinces. The NEPI provided records on hardware inventory and repair requests, which were used to estimate replacement rates and maintenance costs for equipment.

### Data Analysis

#### Overview

This section describes the variables and data analysis approach for each study objective.

For qualitative data analysis, we first transcribed all the interviews. Then, a member of the research team, who was trained on qualitative data methods, was assigned to code the transcripts using Microsoft Excel. This coding process followed a content analysis approach and involved a 3-level coding process: initially, open coding was applied to 5 transcripts to identify major themes; subsequently, the research team held discussions to reach a consensus on the major themes and any emergent themes; and finally, a final codebook was created before coding the remaining transcripts. This approach helped to gain a comprehensive understanding of health workers’ perspectives on improving data quality and data use from the NIIS as well as to identify the barriers and facilitators of immunization coverage.

In the analysis of quantitative data, we used Stata (version 14; StataCorp) as our statistical tool. For categorical variables, we used the chi-square test, and in cases where the expected cell counts were <5, Fisher exact test was used. For continuous variables, we first checked the variable distribution, and given the absence of a normal distribution, the Wilcoxon-Mann-Whitney *U* test was used to compare group differences. In addition, to investigate the relationship between immunization outcomes and its determinants, we conducted a multivariable logistic regression analysis. The threshold for statistical significance was set at *P*<.05.

#### Data Quality and Use

The data use outcome of interest was the percentage of facilities using NIIS data to inform specified routine activities (eg, making monthly vaccination plans).

The data quality outcomes of interest were the quantitative measures of timeliness, completeness, and accuracy. Information from the household and health facility surveys was compared with the NIIS data to assess data quality. Refer to Table S5 in Multimedia Appendix 1 for the definitions of the data quality indicators.

#### Immunization Program Outcomes

Data exported from the NIIS were cleaned. Duplicate records, unreliable data (eg, vaccination date before birth date), and records with “lost to follow-up” status were excluded. The primary outcome of interest was on-time vaccination, determined by the recommended age for vaccine delivery according to the NEPI vaccination schedule [15]. The secondary immunization program outcomes of interest were dropout rates and full vaccination coverage. Multimedia Appendix 1 provides details on NIIS data cleaning and definitions for the primary and secondary outcomes of interest (Table S6 in Multimedia Appendix 1).

#### Costing

Quantitative data were analyzed using Stata (version 14). We computed the total incremental costs per health system level associated with the NIIS implementation. We also estimated the cost per child for the NIIS implementation activities. For this analysis, we allocated a proportion of the costs for NIIS implementation to the 2 provinces according to their annual birth cohort size relative to the national birth cohort. The annual birth cohort for Vietnam in 2019 was approximately 1.5 million [16], whereas the 2 study provinces (Hanoi and Son La) had an annual birth cohort of 162,000 and 25,000, respectively, based on data from the NIIS, representing approximately 12% (187,000/1,500,000) of the annual birth cohort of Vietnam. The NIIS implementation costs were spread over 5 birth cohorts in these cost-per-child calculations, as the system implementation was done over the 5-year period.

To estimate the economic costs associated with service delivery and reporting using either the paper-based system, electronic system, or both, we used an activity-based costing approach. Ingredients or components of the activities were quantified for each resource type, including human resource time use for different immunization activities where there would be a change when using the NIIS versus the paper system, capital costs for equipment and supplies, and recurrent costs for internet connectivity and equipment maintenance attributable to using the NIIS or the paper system. The quantification was done by conducting interviews at the study facilities using structured costing questionnaires. The unit cost of each resource was obtained from secondary data sources, and the total costs for each activity by resource type were estimated. To obtain the total costs, we aggregated the costs for each activity by resource type. We estimated the recurrent costs per facility and per child, with the latter based on only 1 birth cohort as these are annual recurrent costs.

### Ethical Considerations

This study served as the end-line evaluation activity within the project’s work plan. The project was a collaborative effort between the NEPI and PATH. This evaluation constitutes 1 of the project activities outlined in the project documents submitted to the Vietnam Ministry of Health. As per the regulations set forth by the Vietnamese government, the project documents were reviewed and certified by units within the Ministry of Health and other relevant ministries before receiving approval from the Ministry of Health. The study was reviewed, considered as project evaluation, and approved by the Vietnam Ministry of Health (decision 1996/QĐ-BYT), and this study does not require ethics review in accordance with the circular 04/TT-BYT issued by the Vietnam Ministry of Health [17]. This circular [17] regulates the establishment, functions, tasks, and rights of research ethics committees, and it is specified that only research involving human subjects necessitates research ethics committee approval before implementation and supervision during the research process. In addition, the study was reviewed by PATH’s US-based Research Determination Committee, which concluded that the activity did not involve “human subjects” as defined in the US Government 45 Code of Federal Regulations 46.102(e) [18] and did not require US ethics review.

In addition, before conducting the interviews, comprehensive information was provided to all participants, encompassing the study’s objectives, participant rights, and strict confidentiality measures applied to protect their personal information. Informed consent was diligently obtained from each participant before starting the interviews. Their consent to participate in the study was obtained before proceeding with the interviews. Each qualitative interviewee received VN ₫150.000 (US $6.50) as payment for their time. In facilities selected for the costing study, health facility staff received VN ₫400.000 (US $17) for their time participating in structured costing interviews. Interviews were conducted in private settings to ensure confidentiality. All information was coded and only accessible to the study team, and data privacy was emphasized during training of the data collection team. The NEPI provided official permission for the use of the data extracted from the NIIS. All identifying data were coded, and names were eliminated before data analysis.

## Results

### Data Quality and Use

Data quality and use were measured through the NIIS data export, household surveys, and facility surveys and further explained through qualitative interviews.

#### Data Quality

The NIIS data quality evaluation considered the attributes of timeliness, completeness, and accuracy.

#### Timeliness

On the basis of the data exported from the NIIS, timeliness significantly improved from pre- to postintervention for all indicators across all health system levels in both provinces (Table 1). Between pre- and postintervention, there was a significant decrease in the mean number of days from birth date to NIIS registration (Hanoi: 18.6, SD 65.5 to 5.7, SD: 31.4 days; *P*<.001 and Son La: 36.1, SD 94.2 to 11.7, SD 40.1; *P*<.001). Across all health system levels (CHCs, FIFs, and hospitals), there were significant decreases in the mean number of days from the injection date to when the injection was updated in the NIIS. Stock transactions (only assessed at the CHC level) also showed a significant decrease in the mean number of days from stock arrival date to NIIS voucher date (Hanoi: 10.5, SD 36.1 to 5.2, SD: 19.8 days; *P*<.001 and Son La 13.4, SD 38.1 to 6.5, SD 23.5; *P*<.001).

**Table 1:** Timeliness measures pre- and postintervention, broken down by facility type and province.

Level and indicator				Data source		Hanoi				Son La		
					Preintervention		Postintervention	*P* value	Preintervention		Postintervention	*P* value
**Overall**												
		**Registration, n**	N/A^a^		33,861		35,622	N/A	6296		5706	N/A
		Days from birth date to NIIS^b^ registration day^c^, mean (SD)	NIIS data export		18.6 (65.5)		5.7 (31.4)	<.001	36.1 (94.2)		11.7 (40.1)	<.001
**Commune health center level**												
	**Injections, n**		N/A		197,497		148,601	N/A	55,958		48,807	N/A
		Days from injection date to injection updated in the NIIS, mean (SD)	NIIS data export		6.9 (49.1)		2.8 (17.5)	<.001	30.6 (101.4)		4.2 (20.6)	<.001
	**Stock, n**		N/A		76,483		79,573	N/A	12,596		12,168	N/A
		Days from stock arrival date to NIIS voucher date^c^, mean (SD)	NIIS data export		10.5 (36.1)		5.2 (19.8)	<.001	13.4 (38.1)		6. (23.5)	<.001
**Fee-based immunization facility level**												
	**Injections, n**		N/A		202,889		313,117	N/A	1879		5459	N/A
		Days from injection date to injection updated in the NIIS, mean (SD)	NIIS data export		11.5 (64.7)		1.4 (11.7)	<.001	32.3 (98.0)		4.3 (26.0)	<.001
**Hospital Level**												
	**Injections, n**		N/A		27,988		34,698	N/A	5710		7490	N/A
		Days from injection date to injection updated to the NIIS, mean (SD)	NIIS data export		11.6 (66.8)		1.9 (17.1)	<.001	9.0 (58.9)		1.9 (22.9)	<.001

^a^N/A: not applicable.

^b^NIIS: National Immunization Information System.

^c^Dependent variables were not normally distributed; therefore, the Wilcoxon-Mann-Whitney *U* test was used.

#### Completeness

Completeness was assessed by comparing information from the household and facility surveys with the information from the NIIS (Table 2). At the CHC level, completeness of registration, client information, and injection information captured in the NIIS significantly improved between pre- and postintervention in Hanoi and Son La. At the FIF level in Hanoi, there was a decline in the percentage of clients registered in the NIIS; however, among those registered, there was an increase in the completeness of client information. At the FIF level in Son La, there was increased completeness of registration, client information, and immunization information. At the hospital level, there was an improvement in the percentage of clients registered in the NIIS and a decline in the completeness of client information in both provinces, and the completeness of immunization information remained unchanged at 100% (Hanoi: preintervention n=63, postintervention n=153; Son La: preintervention n=41, postintervention n=74) in both provinces at pre- and postintervention.

**Table 2:** Completeness measures pre- and postintervention, by health system level and province.

Level and indicator				Data source		Hanoi						Son La				
						Preintervention		Postintervention	*P* value		Preintervention			Postintervention		*P* value
**Commune health center level**																
	**Registered clients, n**			N/A^a^		229		367	N/A		149			110		N/A
		Clients registered in the NIIS^b^, n (%)	HH^c^ survey		217 (94.8)		362 (98.6)		.007	121 (81.2)			110 (100)		<.001	
	**Client information, n**			N/A		217		362	N/A		119			110		N/A
		Clients with personal information entered fully in the NIIS, n (%)	HH survey		199 (91.7)		359 (99.2)		<.001	101 (84.9)			110 (100)		<.001	
	**Immunization information, n**			N/A		2275		4070	N/A		1322			1106		N/A
		Injections with immunization information entered fully in the NIIS, n (%)	HH survey		2230 (98.0)		4018 (98.72)		.04	1223 (92.51)			1105 (99.90)		<.001	
**Fee-based immunization facility level**																
	**Registered clients, n**			N/A		122		80	N/A		30			20		N/A
		Clients registered in the NIIS, n (%)	FIF^d^ survey		121 (99.2)		72 (90)		.003	27 (90)			(20) 100		.21	
	**Client information, n**			N/A		121		72	N/A		27			20		N/A
		Clients with personal information entered fully in the NIIS, n (%)	FIF survey		117 (96.7)		71 (98)		.65	20 (74)			20 (100)		.02	
	**Immunization information, n**			N/A		121		100	N/A		27			32		N/A
		Injections with immunization information entered fully in the NIIS, n (%)	FIF survey		117 (96.7)		97 (97.0)		.65	20 (74)			32 (100)		.02	
**Hospital level**																
	**Registered clients, n**			N/A		71		100	N/A		43			40		N/A
		Clients registered in the NIIS, n (%)	Hospital survey		65 (91)		100 (100)		.005	41 (95)			39 (97)		.53	
	**Client information, n**			N/A		63		100	N/A		41			39		N/A
		Clients with personal information entered fully in the NIIS, n (%)	Hospital survey		63 (100)		99 (99.0)		.30	41 (100)			38 (97)		.99	
	**Immunization information, n**			N/A		63		153	N/A		41			74		N/A
		Injections with immunization information entered fully in the NIIS, n (%)	Hospital survey		63 (100)		153 (100)		.99	41 (100)			74 (100)		.99	

^a^N/A: not applicable.

^b^NIIS: National Immunization Information System.

^c^HH: household.

^d^FIF: fee-based immunization facility.

#### Accuracy

The accuracy of demographic and immunization information was assessed by comparing information on clients’ personal immunization cards (captured through the household survey) with their information entered in the NIIS. The percentage of clients with demographic information matched between the 2 sources significantly increased in Hanoi (199/217, 91.7% to 340/353, 96.3%; *P*=.02) and Son La (101/119, 84.9% to 107/107, 100%; *P*<.001) from pre- to postintervention (Table 3). The percentage of injections with immunization information matched between the 2 sources also significantly increased in Son La (1037/1216, 85.3% to 1097/1097, 100%; *P*<.001) but significantly decreased in Hanoi (2147/2188, 98.1% to 3786/3981, 95.1%; *P*=.01).

**Table 3:** Accuracy measures pre- and postintervention, by health system level and province.

Level and indicator				Data source		Hanoi							Son La			
						Preintervention		Postintervention		*P* value		Preintervention		Postintervention		*P* value
**Overall**																
	**Demographic information, n**			N/A^a^		217		353		N/A		119		107		N/A
		Clients with demographic information matched between personal immunization card and the NIIS^b^, n (%)	HH^c^ survey		199 (91.7)		340 (96.3)		.02		101 (84.9)			107 (100)	<.001	
	**Immunization information, n**			N/A		2188		3981		N/A		1216		1097		N/A
		Injections with immunization information matched between personal immunization card and the NIIS, n (%)	HH survey		2147 (98.12)		3786 (95.10)		.01		1037 (85.27)			1097 (100)	<.001	

^a^N/A: not applicable.

^b^NIIS: National Immunization Information System.

^c^HH: household.

The interviews indicated that respondents at all levels (national, provincial, district, and facility levels) and across both provinces had a strong understanding of data quality, defined as timeliness, completeness, and accuracy. Most respondents (13/16, 81%) participated in the intervention training on data quality and data use and indicated that it was useful for their work and that they had applied what they learned:

I have participated in the training course on data quality and data use last year. After the training, my knowledge and skill in data quality and data use are better, so I applied to my daily work, I usually check the input data to make them complete and accurate before entering into the system.

CHC staff

All respondents rated their facility’s data quality in the NIIS as “good” or “very good” in terms of timeliness, completeness, and accuracy. All respondents mentioned human resources as the most important factor associated with data quality, including health workers’ knowledge and skills (in data entry, analysis, quality assessment, and use), understanding the importance of data quality, and bandwidth to support the immunization program when working across health areas.

#### Data Use

Data use was measured through facility assessments asking health workers about the activities that the NIIS data were used to inform. In the preintervention survey, 34.8% (202/580) of the facilities in Hanoi and 29.4% (55/187) of the facilities in Son La indicated that they had used the NIIS data for additional activities beyond monthly reporting. In the postintervention survey, 100% (Hanoi: 468/468 and Son La: 199/199) of the facilities in both provinces indicated using the NIIS data for activities beyond monthly reporting. Table S7 in Multimedia Appendix 1 shows the frequency by activity. At postintervention, the most common uses of data among facilities were to inform monthly vaccination plans, campaign plans, or annual immunization plans. At the management level, the most common use of the NIIS data was to evaluate the performance of health facilities. From the qualitative interviews, the most frequently mentioned obstacle to using the data was the lack of health workers’ capacity for data analysis and use.

### Immunization Program Outcomes

On-time vaccination was the primary immunization performance outcome of interest. Secondary outcomes were dropout rates and vaccination coverage (refer to the Tables S8-12 in Multimedia Appendix 1).

#### Study Population Characteristics

Immunization outcomes were assessed by comparing the NIIS data for a cohort of children pre- and postintervention in the 2 provinces. After data cleaning, 81,485 children were included in the sample. Their population characteristics are summarized in Table S8 in Multimedia Appendix 1. In Hanoi, there were small but significant differences in the ethnicity and rural and urban location of children in the pre- and postintervention cohorts. In Son La, there was also a significant difference in the ethnicity of children in the pre- and postintervention cohorts. In both provinces, there were significant differences in the percentage of children vaccinated primarily from FIFs versus CHCs at pre- and postintervention.

#### On-Time Vaccination by Antigen

Across all antigens, apart from the measles-containing vaccine first dose in Hanoi, the percentage of children who received the vaccine on time increased from pre- to postintervention, and nearly all increases were statistically significant (Table 4).

**Table 4:** On-time vaccination coverage, by vaccine and province.

Vaccine	Hanoi				Son La		
	Preintervention (n=33,752), n (%)	Postintervention (n=35,611), n (%)	*P* value	Preintervention (n=6233), n (%)		Postintervention (n=5705), n (%)	*P* value
BCG^a^	29,972 (88.8)	32,400 (90.98)	<.001	3760 (60.32)		4410 (77.3)	<.001
Penta^b^ 1	24,409 (72.31)	31,780 (89.24)	<.001	1935 (31.04)		2985 (52.32)	<.001
Penta 2	8660 (25.66)	13,540 (38.02)	<.001	1999 (32.07)		2810 (49.26)	<.001
Penta 3	8360 (24.77)	10,640 (29.88)	<.001	2169 (34.8)		2695 (47.24)	<.001
Full polio	19,920 (59.02)	26,930 (75.62)	<.001	1965 (31.53)		1855 (32.51)	.28
MCV1^c^	29,900 (88.59)	29,000 (81.44)	<.001	4413 (70.8)		4856 (85.12)	<.001

^a^BCG: bacillus Calmette-Guérin.

^b^Penta: pentavalent.

^c^MCV1: measles-containing vaccine first dose.

In the multiple logistic regression analysis, children at postintervention were approximately 1.18 times as likely in Hanoi and 1.69 times as likely in Son La to receive timely administration of pentavalent (Penta) 3 compared with those in the preintervention cohort (*P*<.001; Table 5). In Hanoi, there was no significant difference in timely Penta 3 vaccination by gender or ethnicity, but children in urban areas were 1.6 times as likely to receive Penta 3 on time compared with those in rural areas. In Son La, there was also no difference by gender, but Thai children were 1.3 times as likely to receive Penta 3 on time compared with other ethnicities. In both provinces, children who were mostly vaccinated from FIFs were more likely to receive Penta 3 on time compared with children mostly vaccinated from CHCs.

A separate logistic regression analysis for on-time full vaccination is included in Table S12 in Multimedia Appendix 1

**Table 5:** Factors associated with Penta 3 timeliness.

Characteristics			Hanoi (n=69,301)					Son La (n=11,918)		
			Odds ratio (95% CI)		*P* value		Odds ratio (95% CI)			*P* value
**Survey round**										
	Preintervention	Reference		N/A^a^		Reference			N/A	
	Postintervention	1.18 (1.13-1.22)		<.001		1.69 (1.56-1.81)			<.001	
**Child’s gender**										
	Girl	Reference		N/A		Reference			N/A	
	Boy	1.0 (0.98-1.04)		.49		1.0 (0.96-1.12)			.32	
**Ethnicity**										
	Kinh	1.1 (0.83-1.44)		.52		1.1 (0.98-1.20)			.12	
	Thai	0.7 (0.28-1.78)		.46		1.3 (1.21-1.44)			<.001	
	Other ethnicities	Reference		N/A		Reference			N/A	
**Region**										
	Rural	Reference		N/A		Reference			N/A	
	Urban	1.6 (1.51-1.62)		<.001		N/A			N/A	
**Vaccinated mostly from**										
	Commune health center	Reference		N/A		Reference			N/A	
	Fee-based facility	2.7 (2.62-2.82)		<.001		1.59 (1.1-2.3)			.01	

^a^N/A: not applicable.

#### On-Time Full Immunization Coverage

**Figure 1:**
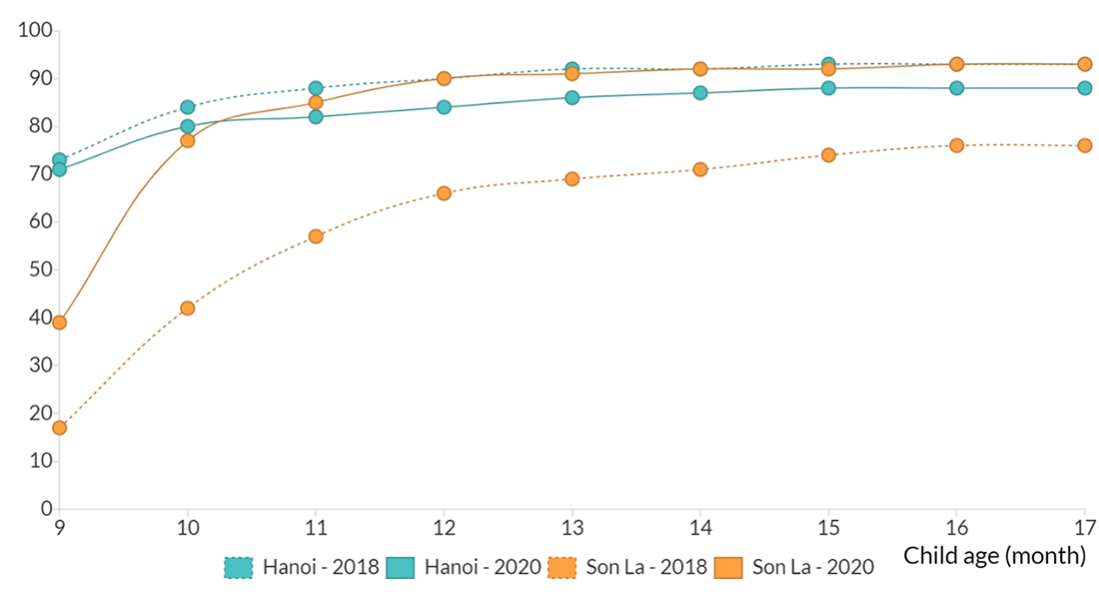
Full immunization coverage by age for the pre- (2018) and postintervention (2020) birth cohorts, by province.

Figure 1 shows the full immunization coverage over time for each birth cohort. In Hanoi, 87.8% (29,649/33,752) of the children in the preintervention cohort and 81.9% (29,167/35,611) of the children in the postintervention cohort (*P*<.001) had reached full immunization before their first birthday. In contrast, there was an increase in the percentage of children reaching full immunization before their first birthday in Son La, from 63.3% (3947/6233) preintervention to 88.3% (5040/5705) postintervention (*P*<.001). The results for immunization coverage by antigen for the pre- and postintervention birth cohorts in each province are included Table S9 in Multimedia Appendix 1.

### Costs

#### Up-Front Costs of NIIS Implementation

The up-front NIIS software design, development, and deployment took approximately 2300 person-months of labor from 2015 to 2020, and the partner costs for this labor were estimated at approximately US $1.75 million (Table 6). Most of the software developer costs (US $1,233,152/US $1,745,712, 71%) pertained to the deployment of the system. In addition, there were up-front costs per facility for implementing the NIIS, including costs for back entry of data, internet setup (where needed), training of users, and meeting costs. At the national and provincial levels, the bulk of the up-front costs were spent on training and meetings. As trainings were paid for by the higher administrative levels, training costs are low or 0 at district levels and health facilities. At these levels, the larger cost share was for deploying the NIIS.

**Table 6:** Estimated up-front financial costs of implementing the NIISa between 2015 and 2020.

Level and indicator				Amount (US $)
**National level, total**				
	**Software development**			
		NIIS design	198,457	
		NIIS development	314,103	
		NIIS deployment	1,233,152	
	Up-front costs			1,745,712
	Training and deployment			53,911
**Subnational levels, mean (SD)**				
	**Provincial Centers for Diseases Control and Prevention (costs per province; n=2)**			
		Deployment and training	41,259 (41,830)	
	**District health centers (costs per district; n=12)**			
		Deployment and training	762 (402)	
	**Commune health centers (costs per commune health center; n=24)**			
		Deploying NIIS	69 (61.7)	

^a^NIIS: National Immunization Information System.

The up-front costs for the NIIS implementation allocated to the 2 study provinces were approximately US $419,000 (Table 7). When these costs are allocated over 5 birth cohorts, the estimated cost per child for the NIIS implementation was estimated at US $0.48.

**Table 7:** Incremental financial cost for developing and deploying the NIIS^a^ allocated to the 2 study provinces.

Cost classification	Amount (US $)
System design, development, and deployment costs by the software developer	217,726
Trainings and deployment costs by NEPI^b^ and subnational levels	201,061
Total cost allocated to the 2 study provinces	418,787

^a^NIIS: National Immunization Information System.

^b^NEPI: National Expanded Program on Immunization.

#### Recurrent Costs for the NIIS

The software developer estimated the annual recurrent costs for the system operation to be US $85,000. At health facilities, the average monthly economic cost for health worker labor for immunization-related activities done using the paper system were estimated to be US $146 (Table 8). In comparison, the monthly cost for labor with the NIIS was US $67, which is less than half of the labor cost when using the paper system. The most significant savings in labor time costs, resulting from the transition from the paper system to the NIIS, occurred through reduced time spent on organizing immunization sessions, data management, and reporting. However, the NIIS also resulted in additional activities for staff, including checking of duplicates and introduction of e-immunization cards. The NIIS also added new facility costs, including recurrent costs for internet, printing, and SMS text messaging reminders and the capital costs of equipment, which amounts to an average cost of US $28 per facility. However, with the NIIS, there were savings in printing costs as registers and ledgers would not be printed, and the average costs of these were US $4.51 per month or US $58 per year. Overall, the total monthly costs per facility with NIIS (US $95) are lower than with the paper system (US $151).

**Table 8:** Estimated recurrent economic costs per facility for the paper-based system compared with the NIIS^a^ at the commune health centers (n=24).

Health facilities		Cost per facility (US $), mean (SD)	Estimated cost (US $), IQR
**Monthly cost of the paper system**			
	Labor cost for health workers	146 (15.1)	23-155
	Printing of registers and stock ledgers	4.51 (1.7)	4.51-4.51
Total monthly cost of the paper system		151 (15.2)	28-160
**Monthly cost of the NIIS**			
	Recurrent cost	2 (1.1)	01-03
	Equipment cost	26 (4.1)	19-33
	Labor cost for health workers	67 (36.9)	15-86
Total monthly cost of the NIIS		95 (35.3)	35-122

^a^NIIS: National Immunization Information System.

Table 9 presents similar costs for the administrative levels. At most levels, except at provincial CDC, labor costs are lower with the NIIS than with the paper system. At the CDC, the labor costs for supportive supervision are the largest share of costs, and these costs increase when using the NIIS compared with when using the paper system. At the district health centers, there is a decline in labor costs for activities such as management and reporting when using the NIIS, and hence, labor costs are lower with the NIIS. As mentioned above, implementing the NIIS incurs additional costs, including recurrent costs for internet, printing, and equipment maintenance and capital costs for equipment, which makes the total monthly costs for NIIS more than the total monthly costs for the paper system. There are incremental costs resulting from the change from the paper system to the NIIS at the administrative levels.

**Table 9:** Estimated economic costs per district, province, and at the national level for the paper-based system compared with the NIIS^a^.

Level and cost			Cost per facility (US $), mean (SD)	Estimated cost (US $), IQR
**District health centers (n=12)**				
	**Monthly cost of the paper system**			
		Labor cost for health workers	74 (35.5)	17-88
		Printing of registers and stock ledgers	4.51 (1.2)	4.51-451
		Total monthly cost of the paper system	79 (36)	22-93
	**Monthly cost of the NIIS**			
		Recurrent cost	296 (141.4)	153-419
		Equipment cost	46 (11.5)	32-62
		Labor cost for health workers	61 (32.2)	10-63
		Total monthly cost of the NIIS	403 (151.4)	195-543
**Provincial Centers for Disease Control and Prevention (n=2)**				
	**Monthly cost of the paper system**			
		Labor cost for health workers	212 (130)	74-350
		Printing of registers and stock ledgers	4.51 (1.7)	4.51-451
		Total monthly cost of the paper system	217 (130.3)	79-355
	**Monthly cost of the NIIS**			
		Recurrent cost	1 (0.09)	1-1
		Equipment cost	148 (13)	132-163
		Labor cost for health workers	300 (235.5)	73-526
		Total monthly cost of the NIIS	449 (224.9)	206-690
**National Expanded Program on Immunization**				
	**Monthly cost of the paper system**			
		Labor cost for health workers	320 (113.1)	N/A^b^
		Printing of registers and stock ledgers	4.51 (0.7)	N/A
		Total monthly cost of the paper system	325 (113.8)	N/A
	**Monthly cost of the NIIS system**			
		Recurrent cost	152 (4.2)	N/A
		Equipment cost	15 (0)	N/A
		Labor cost for health workers	287 (18.4)	N/A
		Total monthly cost of the NIIS	455 (22.6)	N/A

^a^NIIS: National Immunization Information System.

^b^N/A: not applicable.

Although labor costs decrease at most health system levels with the transition to the NIIS, in practice, this may not translate into budget line savings as staff are retained and their time is reallocated. The financial costs associated with the paper system primarily entail printing registers, but these expenses are relatively minor in comparison with the those incurred with the electronic system. The estimated annual financial recurrent costs per child for the NIIS are US $3.17, and these account for the annual costs of the server (with the relevant portion allocated to the study provinces) and the costs for capital equipment and internet. There are incremental financial costs to the health system when moving from the legacy paper system to the electronic system.

## Discussion

### Principal Findings

Health workers at the province, district, and CHC levels successfully transitioned from the legacy paper-based system to the NIIS for paperless reporting, proving that the transition was possible in 2 very different provincial contexts. Although the transition to paperless reporting results in lower labor costs at the facility, district, and national levels, it requires incremental financial recurrent costs (estimated at US $3.17 per child per year) to maintain the NIIS.

This study found improvements in data quality in Hanoi and Son La between pre- and postintervention. There were significant improvements in data timeliness at all levels of the health system and in both provinces. It is likely that the interventions contributed to this improvement because the guidelines, trainings, data review meetings, and supportive supervision visits emphasized the importance of timely data. Timeliness indicators performed better in Hanoi than in Son La, which may be because of the different service delivery practices; in Hanoi, vaccines are delivered on a weekly basic, whereas in Son La, they are delivered monthly. In addition, in Hanoi, more clients visit FIFs that deliver immunization daily.

In Son La, there were also improvements across nearly all the completeness and accuracy indicators. However, there were mixed results in terms of data completeness and accuracy in Hanoi, with some statistically significant declines in quality. This may be partly because Hanoi was more affected by the COVID-19 pandemic than Son La; there were 6 rounds of mandated social distancing across all 30 districts in Hanoi. When immunization services were available, facilities may have been understaffed if human resources were reassigned to the COVID-19 response and they may have faced higher demand from a backlog of children who could not be vaccinated during social distancing. Overburdened health workers may prioritize service delivery over data quality.

The observed differences in provincial results may also have been influenced by the way interventions were delivered in each province. The facilities in Son La received timely support through internet-based supervisory assistance. In Hanoi, they maintained in-person supportive supervision, which was less timely, and limited the number of facilities that could be visited. Facilities in Son La also received refresher trainings, which Hanoi did not, as Hanoi had an ongoing focus on COVID-19 vaccination.

There were large increases from pre- to postintervention in the percentage of facilities using the NIIS data to inform their activities. Results from the costing analysis showed that at the CHCs, health workers’ time spent on immunization program data entry and reporting declined as the transition to paperless resulted in efficiencies and reduced workload. It is possible that this saved time was dedicated to data use.

For most activities, a higher percentage of facilities in Son La reported using NIIS data compared with Hanoi. These results may have also been influenced by the COVID-19 pandemic or the differences in project interventions. In addition, Hanoi has a more mobile population, with clients frequently moving between facilities. This implies that vaccination plans informed by NIIS data may not be as accurate or comprehensive compared with a location such as Son La, where there is less population movement.

Although only 44.7% (84/188) of facilities in Son La and 27.7% (125/451) of facilities in Hanoi reported using the NIIS to evaluate data quality, previous studies have shown that data use can drive data quality improvements [19]. Through the qualitative interviews, health workers recommended building data quality indicators directly into the NIIS to help users understand their data quality easily.

The study also found that the majority of on-time vaccination rates for various antigens in the 2 project provinces improved compared to those in the preintervention group. With more timely data captured in the NIIS, health workers know which vaccines a child is due for and can send reminders or follow-up to deliver the vaccines on time. For the clients using the e-immunization card, it may have also contributed to timely vaccination by reminding clients of their vaccination schedule and due dates. However, the on-time vaccination rate for the first dose of measles in Hanoi decreased after the intervention compared to before, which may have been owing to the COVID-19 lockdowns from July to September 2021, when the postintervention cohort was aged 9 to 12 months. In Hanoi, many parents prefer waiting to receive the measles-rubella vaccine at 12 months, which is delivered at FIFs, versus the monovalent measles vaccine, which is part of the standard EPI schedule at 9 months.

In Son La, improvements in data quality and on-time vaccination were also associated with improvements in vaccination coverage. Full immunization at 12 months was 88.3% (5040/5705) in the postintervention cohort compared with 63.3% (3947/6233) in the preintervention cohort. A previous evaluation of ImmReg (the earlier version of the NIIS) in another province in Vietnam, Ben Tre, also found improvements in on-time vaccination and vaccination coverage when comparing pre- and postintroduction of the digital immunization registry, and these improvements were sustained or increased 1 year later [6]. However, in Hanoi, despite improvements in on-time vaccination, there were declines in vaccination coverage, again likely because of the COVID-19 pandemic. According to the 2021 annual EPI report for Vietnam, full immunization coverage decreased nationwide from 96.8% in 2020 to 87.1% in 2021 [20].

Introducing these new electronic systems, which have improved data quality and timeliness and could potentially improve immunization outcomes, comes at a cost, as up-front financial investments have to be made for software design, development, and deployment. The cost per child for NIIS development and deployment was estimated at US $0.48 in the 2 study provinces in Vietnam. This is much lower than that estimated in Tanzania and Zambia, the only other low- and middle-income countries with comparable cost estimates for the development of electronic immunization registries [11,12]. In our study, costs were annualized over 5 birth cohorts, whereas in Tanzania and Zambia, costs were annualized over 3 birth cohorts. Even if we were to use 3 birth cohorts for this analysis, our estimated costs for Vietnam would still be lower. The estimated lower costs per child for Vietnam could be in part because most CHCs had existing computers, and hence, there was no mass procurement of equipment required, which is not the case in many other countries. In Vietnam, the equipment and connectivity costs were shared across multiple health programs, further reducing the costs borne by the immunization program, unlike in Tanzania and Zambia, where immunization was the first health area to be digitalized at the health facility level. The availability of electricity at the health facility level in Vietnam also reduced costs, as there was no need for the procurement of alternative power sources such as solar chargers. In addition, software development in Vietnam was conducted by an in-country telecommunications partner that provided in-kind and pro bono services and support, some of which could not be quantified, resulting in lower costs than if done through an external organization.

Our study found that at the CHC level, there are cost savings for health workers’ time use and other resources such as printing with the NIIS compared with the paper system, and at the administrative level, there are incremental costs. The NIIS results in some savings in labor time, although some new activities are added for the staff. There are also recurring costs, such as ongoing technical support and maintenance provided by Viettel, the need for refresher training at the local level for new staff, and expenses associated with equipment maintenance and replacement as well as connectivity provided by local government. These recurrent costs are important to consider for sustainability.

### Limitations

Our study has several limitations. First, we conducted the study in 2 provinces and had small sample sizes for the costing results, facility survey results for data quality, and other results based on provider interviews. The small sample size and purposive sampling limit the generalizability of our findings; however, 2 distinct provinces with a mix of facility types were intentionally sampled so that the results can be used to understand the costs and outcomes of the transition to paperless reporting in a range of settings. Second, the time frame for the evaluation was short, which limited the magnitude of the change that could be observed. Third, there were minor differences in the data collection approaches and responses between pre- and postintervention. For example, the urban sampling approach was adjusted for the feasibility of data collection, and the response rate for facility assessment was lower at postintervention. Fourth, for data collected through provider interviews, such as for the costing study, potential recall bias arises due to retrospective nature of the inquires, as interviewees were asked to recall cost from past periods. Future studies should consider including costing data collection at different phases of the project to reduce recall bias. This would also facilitate the availability of costing data available to inform decision-making at various implementation phases. Our study may also have underestimated the costs associated with the paper system, as we did not incorporate expenses related to printing paper home-based records and invitation letters. These materials are printed to assist the caregivers in monitoring their children’s vaccination history or reminding them when their child is due for vaccination. We also have not accounted for the saved labor costs for health care workers who would deliver the invitation letters to households. Another limitation is that the COVID-19 pandemic affected the originally planned timelines for data collection and the immunization service delivery. We did not try to account for the impact of the pandemic on findings regarding the data quality and data use presented in this study.

### Conclusions

Health workers in the 2 provinces successfully transitioned to paperless reporting while maintaining or improving data quality. We recommend that other provinces in Vietnam transition to paperless reporting by introducing the guidelines and standard operating procedures used in Hanoi and Son La and providing ongoing support through trainings, data review meetings, supportive supervision, and peer networks. Future studies should monitor data quality and immunization outcomes in other provinces as well as the sustainability of the observed changes in Hanoi and Son La.

Introducing these new electronic systems comes with costs—both up-front and recurrent—but there are advantages, as seen in the improvements in data quality and on-time vaccination. In Vietnam, stakeholders should plan and budget for the sustainability of the system at each level of the health system, given the recurrent costs including repairing and replacement of equipment, connectivity, refresher training, software system, and supportive supervision. Other countries planning to implement similar interventions should plan to collect costing data throughout to inform decision-making and budgeting.

## Appendix

Multimedia Appendix 1 Supplementary tables.

## Abbreviations

CDC: Centers for Disease Control and Prevention
CHC: commune health center
EPI: Expanded Program on Immunization
FIF: fee-based Immunization facility
NEPI: National Expanded Program on Immunization
NIIS: National Immunization Information System
Penta: Pentavalent

## Data Availability

The data sets generated during and analyzed during this study are available from the corresponding author on reasonable request.
