# Supplementary material for: Outcomes and Costs of the Transition From a Paper-Based Immunization System to a Digital Immunization System in Vietnam: Mixed Methods Study
Source: J Med Internet Res. 2024 Mar 18;26:e45070. doi: 10.2196/45070 (PMC10985597; doi:10.2196/45070)
Supplement: Multimedia Appendix 1 [file jmir_v26i1e45070_app1.docx]

**Supplementary materials**

Methods

1. Overview of characteristics of the study provinces
2. COVID-19 social distancing in the study provinces
3. Summary of data sources and data collection methods
4. List of communes, districts, and facilities sampled
5. Data quality indicator definitions
6. Details on the National Immunization Information System (NIIS) data export
7. Definition for primary immunization program outcome of interest
8. Definitions for secondary immunization program outcomes of interest

Results

1. Data use results
2. Study population characteristics
3. Full immunization coverage results
4. Immunization dropout results
5. Factors associated with on-time full immunization
6. **Overview of characteristics of the study provinces**

Table S1. Characteristics of the study provinces.

| **Characteristics** | **Hanoi** | **Son La** | **Total** |
| --- | --- | --- | --- |
| **Number of districts** |  |  |  |
| Urban district | 12 | 1 |  |
| Rural district | 18 | 11 |  |
| **Number of communes** |  |  |  |
| Urban commune | 166 | 1 |  |
| Rural commune | 413 | 1 |  |
| Mountainous commune | 0 | 202 |  |
| **Mean number of villages per commune** | 9.1 | 12.3 |  |
| **Number of fee-based facilities** | 210 | 20 |  |
| **Number of hospitals have birth rooms** | 51 | 13 |  |
| **Mean number of staff per commune heath center** | 6.1 | 22.7 |  |
| **Number of children under 1 year of age** | 134,391 | 20,911 |  |
| **Number of children under 5 years old** | 756,584 | No information |  |
| **Distance from CHC to center of district (km)** | 5.5 | 25 |  |
| **Distance from village to CHC (km)** | 2 | 8 |  |

1. **COVID-19 social distancing in the study provinces**

Table S2. Dates that COVID-19 social distancing was applied.

| **Province** | **Time** | | **# days** | **# districts** |
| --- | --- | --- | --- | --- |
|  | **From** | **To** |  |  |
| Son La | 13 August 2021 | 1 September 2021 | 20 days | 1/12 districts |
|  | 2 September 2021 | 14 September 2021 | 15 days | 1/12 districts |
|  | 24 September 2021 | 3 October 2021 | 10 days | 1/12 districts |
| Hanoi | 1 April 2020 | 15 April 2020 | 15 days | 30/30 districts |
|  | 16 April 2020 | 22 April 2020 | 7 days | 30/30 districts |
|  | 24 July 2021 | 7 August 2021 | 15 days | 30/30 districts |
|  | 8 August 2021 | 22 August 2021 | 16 days | 30/30 districts |
|  | 23 August 2021 | 6 September 2021 | 15 days | 30/30 districts |
|  | 7 September 2021 | 21 September 2021 | 16 days | 30/30 districts |

1. **Summary of data sources and data collection methods**

A summary of data collection methods across the three study aims (data quality and use, immunization performance, and costing) is presented in Table S3.

Table S3. Summary of data sources, collection methods, timing, and sampling.

| **Study aim** | **Data sources / data collection methods** | **Sampling unit and sample size** | **Timing of data collection** (fieldwork completed) | **Information captured** |
| --- | --- | --- | --- | --- |
| Data quality and use | Self-administered facility assessment sent via email | All immunization facilities in Hanoi and Son La Provinces were sent assessments. Assessments were completed by:  Pre: 990 facilities (747 in Hanoi, 243 in Son La)  Post: 707 facilities (500 in Hanoi, 207 in Son La). | Pre: July 2019  Post: November 2021 | Basic information about infrastructure, capacity, human resources, and NIIS data use. |
| Data quality and use | Household survey | Pre: Households with children born from 01 July 2017 to the day of data collection in 18 selected communes (12 in Hanoi; 6 in Son La).  Post: Households with children born from 01 January 2019 to the day of the survey in the same 18 communes. | Pre: July 2019  Post: November 2021 | Demographic and immunization information from home-based records (for comparison with the NIIS). |
| Data quality and use | Fee-based immunization facilities (FIFs) and hospitals survey | Pre: 8 FIFs and 7 hospitals.  Post: 5 FIFs and 7 hospitals.  In each FIF/hospital, 20 clients in the paper logbook were randomly selected. | Pre: July 2019  Post: November 2021 | Demographic and immunization information from 20 clients randomly selected from the paper logbook (for comparison with the NIIS). |
| Data quality and use; immunization program outcomes | Data exported from the NIIS | Pre: All children born from 01 July to 30 Sept. 2018 registered in the NIIS.  Post: All children born from 01 July to 30 Sept. 2020 registered in the NIIS. | N/A | Demographic and immunization information from all children in the birth cohort to assess data quality and immunization timeliness, coverage, and drop-out rates. |
| Data quality and use; immunization program outcomes | In-depth interviews with EPI officers | Post: 16 interviews. | Post: November 2021 | Factors related to data quality, data use, and immunization program outcomes. |
| Costing | Record review and interviews with EPI officers using structured costing questionnaires | Cross-sectional surveys at 12 district health centers, 24 commune health centers, and 11 hospitals. | November 2021 | Expenditures and quantities of resources used for NIIS or paper-based system. |

1. **List of communes, districts, and facilities sampled**

Facility surveys were conducted in a purposively selected sample of districts, communes, hospitals, and fee-based immunization facilities (FIFs) in each province, identified in consultation with NEPI and provincial CDCs. Five hospitals in Hanoi and two hospitals in Son La were selected to represent urban, rural, private, public at different levels. Six FIFs were selected in Hanoi, and two FIFs were selected in Son La. In each facility, 20 clients in the paper logbook were randomly selected, and their demographic and immunization information were captured. A full list of facilities included in the different evaluations conducted as part of this study is included in Table S4.

A household survey was conducted in a sample of households with children under the age of 2 years to capture demographic and immunization information about the children from their home-based immunization cards. The household survey was conducted in a purposively selected sample of districts and communes. In Hanoi, 6 of the 30 districts were selected to represent a mix of facility types (fee-based, private, public) and geographies (urban, semi-urban, rural). In Son La, 3 of the 12 districts were selected to represent urban, rural, and mountainous areas. Within each district, two communes were selected: one with smooth operation of the National Immunization Information System (NIIS) and one with challenges. In Son La and in rural communes in Hanoi, one village was selected for convenience in each commune, and all households with children under the age of 2 years were included. In urban communes in Hanoi, a living quarter was selected for convenience, the estimated children in the age range were mapped, and two buildings with approximately 80 households were randomly selected; all households with children under the age of 2 years in the selected buildings were included.

The data collection samples were the same for pre- and post-intervention timepoints except for a minor change in the household survey sampling. The household survey purposive sampling for urban households in Hanoi was adjusted from 30-clusters sampling (pre) to randomly select two buildings in a living quarter (post); this approach improved the feasibility of data collection and allowed the team to select a subset of buildings with mixed populations.

Table S4. List of facilities included in the different study components by province.

| **Facility type** | **Ha Noi** | | | | **Son La** | | | |
| --- | --- | --- | --- | --- | --- | --- | --- | --- |
|  | **Name** | **Data quality & use** | **Costing** | **EPI interview** | **Name** | **Data quality & use** | **Costing** | **EPI interview** |
| District health center | Chuong My |  | X |  | Moc Chau |  | X |  |
|  | Phuc Tho |  | X | X | Quynh Nhai |  | X | X |
|  | Thanh Oai |  | X |  | Son La City |  | X | X |
|  | Ha Dong |  | X |  | Mai Son |  | X |  |
|  | Hai Ba Trung |  | X | X | Bac Yen |  | X |  |
|  | Long Bien |  | X |  | Song Ma |  | X |  |
| Commune health center | Quang Bi | X | X |  | Chieng Hac | X | X | X |
|  | Tan Tien | X | X | X | Dong Sang | X | X |  |
|  | Vong Xuyen | X | X |  | Chieng on | X | X |  |
|  | Tho Loc | X | X |  | Muong Giang | X | X | X |
|  | Bich Hoa | X | X |  | Chieng An | X | X | X |
|  | Kim Bai | X | X | X | Chieng Le | X | X |  |
|  | Bien Giang | X | X |  | Chieng Mung |  | X |  |
|  | Phuc La | X | X | X | Chieng Ve |  | X |  |
|  | Thanh Nhan | X | X |  | Phieng Ban |  | X |  |
|  | Vinh Tuy | X | X |  | Hong Ngai |  | X |  |
|  | Cu Khoi | X | X | X | Song Ma town |  | X |  |
|  | Duc Giang | X | X |  | Huoi Mot |  | X |  |
| Fee-based immunization facility (FIF) and hospital | 131 Lo Duc (FIF) | X | X | X | Thao Nguyen Hospital | X | X |  |
|  | Vinmec Hospital (FIF) | X | X |  | Moc Chau (FIF) |  | X |  |
|  | Hai Ba Trung (FIF) |  | X |  | Quynh Nhai Hospital | X | X |  |
|  | Vinmec Hospital |  | X |  | Quynh Nhai (FIF) | X | X | X |
|  | National institute for control of vaccine and biologicals (FIF) | X | X |  | Provincial Hospital | X | X |  |
|  | Buu Dien Hospital | X | X | X | Life General Hospital |  | X |  |
|  | Dong Anh Hospital (FIF) |  | X |  | Mai Son (FIF) |  | X |  |
|  | Dong Anh Hospital | X | X | X |  |  |  |  |
|  | Thu Cuc Hospital (FIF) |  | X | X |  |  |  |  |
|  | Thu Cuc Hospital | X | X |  |  |  |  |  |
|  | National Hospital of Obstetrics and Gynecology | X | X |  |  |  |  |  |
|  | Hanoi Hospital of Obstetrics and Gynecology | X | X |  |  |  |  |  |
|  | Duong Noi (FIF) |  | X |  |  |  |  |  |
|  | VNVC (FIF) | X | X |  |  |  |  |  |

1. **Data quality indicator definitions**

Table S5. Data quality indicator definitions

| **Data quality attribute** | **Health system level** | **Indicator** | **Data source** | **Numerator** | **Denominator** | **Comments** |  |
| --- | --- | --- | --- | --- | --- | --- | --- |
| Timeliness | Overall | Mean days from birth date to NIIS registration day | NIIS data export | total # of days from birth date to registration date in the NIIS for all children registered in the NIIS  (where birth date = day 0) | total number of children |  |  |
| Timeliness | CHC | Mean days from injection date to injection updated in the NIIS | NIIS data export | total # of days from injection date to updated date in the NIIS for injections delivered at CHCs  (where injection date = day 0) | total # of injections updated into the system |  |  |
| Timeliness | FIF | Mean days from injection date to injection updated in the NIIS | NIIS data export | Total # of days from injection date to updated date in the NIIS for injections delivered at FIFs  (where injection date = day 0) | total # of injections delivered at FIFs |  |  |
| Timeliness | Hospital | Mean days from injection date to injection updated in the NIIS | NIIS data export | total # of days from injection date to updated date in the NIIS for injections delivered at hospitals  (where injection date = day 0) | total # of injections delivered at hospitals |  |  |
| Timeliness | CHC | Mean days from stock arrival date to NIIS voucher date | NIIS data export | total # of days from date of stock arrival/vaccine dispatch to the date created on the NIIS  (where stock arrival date = day 0) | total # of vouchers | Vaccines and supplies are transferred from the district to CHCs and all CHC stock transactions should be captured in the NIIS. This indicator was not measured at FIFs because they have a separate supply chain for vaccines that does get captured in the NIIS, and it was not measured at hospitals since they only deliver birth doses. |  |
| Completeness | CHC | % of clients registered in the NIIS | Household survey, NIIS | # of clients identified in the household survey who are registered in the NIIS | Total # of clients identified in the household survey |  |  |
| Completeness | FIF | % of clients registered in the NIIS | FIF survey, NIIS | # of clients identified in the FIF survey who are registered in the NIIS | Total # of clients identified in the FIF survey | In each FIF, 20 clients in the paper logbook were randomly selected to compare the data of clients recorded in the log-book with the data in the NIIS. |  |
| Completeness | Hospital | % of clients registered in the NIIS | Hospital survey, NIIS | # of clients identified in the Hospital survey who are registered in the NIIS | Total # of clients identified in the Hospital survey | In each hospital, 20 clients in the paper newborn registry were randomly selected to compare the data of clients recorded in the paper registry with the data in the NIIS. |  |
| Completeness | CHC | % of clients with personal information entered fully in the NIIS | Household survey | # of clients identified in the household survey who are registered in the NIIS with complete demographic information | total # of clients with data collected through the household survey AND who are registered in the NIIS | Complete demographic information includes full name, gender, date of birth, and address |  |
| Completeness | FIF | % of clients with personal information entered fully in the NIIS | FIF survey, NIIS | # of clients identified in the FIF survey who are registered in the NIIS with complete demographic information | total # of clients with data collected through the FIF survey AND who are registered in the NIIS | Complete demographic information includes full name, gender, date of birth, and address |  |
| Completeness | Hospital | % of clients with personal information entered fully in the NIIS | Hospital survey, NIIS | # of clients identified in the hospital survey (newborn registry) who are registered in the NIIS with complete demographic information | total # of clients with data collected through the hospital survey AND who are registered in the NIIS | Complete demographic information includes full name, gender, date of birth, and address |  |
| Completeness | CHC | % of injections with immunization information entered fully in the NIIS | Household survey, NIIS | # of injections identified in the household survey with complete injection information entered in the NIIS | total # of injections identified in the household survey | Complete injection information includes the name of vaccines, doses, and dates of injection | |
| Completeness | FIF | % of injections with immunization information entered fully in the NIIS | FIF survey, NIIS | # of injections identified in the FIF survey with complete injection information entered in the NIIS | total # of injections identified in the FIF survey | Complete injection information includes the name of vaccines, doses, and dates of injection |  |
| Completeness | Hospital | % of injections with immunization information entered fully in the NIIS | Hospital survey, NIIS | # of injections identified in the hospital survey (newborn registry) with complete injection information entered in the NIIS | total # of injections identified in the hospital survey | Complete injection information includes the name of vaccines, doses, and dates of injection |  |
| Accuracy | Overall | % of clients with demographic information matched between personal immunization card and the NIIS | Household survey, NIIS | # of clients with data collected through the household survey AND who are registered in the NIIS with demographic information (full name, gender, date of birth, address) matched between personal immunization card and the NIIS | # of clients with data collected through the household survey AND who are registered in the NIIS | Among those clients captured in both the HH survey and NIIS, this indicator analyzes alignment of their demographic information. Their demographic information matches if they have the same full name, gender, date of birth, and address on their personal immunization card and in the NIIS. |  |
| Accuracy | Overall | % of injections with immunization information matched between personal immunization card and the NIIS (doses and date of injection) | Household survey, NIIS | # of injections immunization registered in personal immunization card and the NIIS information matched between both sources | total # of injections registered in personal immunization card and the NIIS | Immunization information matches if it captures the same dose and date of injection. |  |

1. **Details on the NIIS data export**

Initially, data for 92,344 children who were included in the pre-/post-intervention sampling cohorts were extracted from the NIIS. This corresponded to 1,385,931 records, where each record is a vaccine event. The figure below explains why some records and/or children were excluded in the data cleaning process. Records and/or children were excluded if they had a “lost to follow up” status (referred to in the figure below as ‘no monitoring category’), if their vaccinated data was sooner than their date of birth, or if they were a duplicate case. The final cross-sectional sample included 81,301 children.

1. **Definition for primary immunization program outcome of interest**

The primary outcome of interest was on-time vaccination, determined by the recommended age for vaccine delivery. In Vietnam, NEPI vaccination service is conducted on fixed days each month (except for Hanoi with weekly immunization), so if a child was born one day after the last day of monthly vaccination service, this child will have to wait one more month to receive the vaccine. Therefore, the Vietnam NEPI and this study define on-time vaccination as presented in Table S6.

Table S6. National EPI vaccination schedule for bacillus Calmette-Guérin, pentavalent, polio, and measles-containing vaccine first dose (MCV1) vaccines and on-time range for analysis.

| **Recommended schedule with the National EPI program** | **Vaccine** | **On-time range** |
| --- | --- | --- |
| At birth (as soon as possible within the first 30 days) | BCG | 0–30 days after birth |
| 2 months | Penta 1 | 56–89 days after birth |
| 3 months | Penta 2 | 28–31 days after receiving the first shot |
| 4 months | Penta 3 | 28–31 days after receiving the second shot |
|  | Full polio^a^ | 119–178 days |
| 9–11 months | MCV1 | 270–330 days |

^a^Full polio: Three doses of polio (including at least one dose of inactivated polio vaccine).

Abbreviations: BCG, bacillus Calmette-Guérin; MCV1, measles-containing vaccine first dose; Penta, pentavalent.

1. **Definitions for secondary immunization program outcomes of interest**

Secondary immunization program outcomes of interest were drop-out rates and full vaccination coverage. The drop-out rate was calculated as the difference in coverage between OPV1 vs. OPV3, DPT1 vs. DPT3, and BCG vs. MCV1. The drop-out rate was calculated by the following formula:

| *# of children administrated dose 1 - # of children administrated dose 3* | *X 100%* |
| --- | --- |
| *# of children administrated dose 1* |  |

Full vaccination coverage, as defined by NEPI, is when a child has received his or her eight childhood vaccines: Bacillus Calmette-Guérin (BCG), three doses of hepatitis B, three doses of DPT, three doses of Hib, three doses of polio (including at least one dose of IPV), and one dose of measles containing vaccine (MCV1). On-time full immunization coverage is then defined as the number of children who are fully immunized with all of these basic vaccines before their first birthday, divided by the total number of surviving children who were born in the same period.

1. **Data use results**

Table S7. Percent of facilities self-reporting using NIIS data at post-intervention, by activity, health system level, and province.

| **Activity using NIIS data** | **Percent of facilities using NIIS data** | | |
| --- | --- | --- | --- |
| **Management level (district health centers and provincial CDC)** | **Hanoi**  **(n=17)** | **Son La**  **(n=11)** |  |
| Evaluate the performance of health facilities | 88.2 | 100 |  |
| Evaluate the data quality | 64.7 | 100 |  |
| Identify priority of supportive supervision | 58.8 | 90.9 |  |
| Track the defaulters/drop-out rate | 82.4 | 90.9 |  |
| Identify the reasons and provide evidence for decision-making | 47.1 | 81.8 |  |
| Make annual immunization planning | 76.5 | 72.7 |  |
| Make monthly vaccination plan | 70.6 | 72.7 |  |
| Identify the low proportion of DPT3 | 70.6 | 72.7 |  |
| Make plan for campaign vaccination | 64.7 | 63.6 |  |
| Using NIIS data for other programs | 41.2 | 63.6 |  |
| Make plan for vitamin A program | 23.5 | 27.3 |  |
| **For facilities (CHCs, FIFs, and hospitals)** | **Hanoi**  **(n=451)** | **Son La**  **(n=188)** |  |
| Make monthly vaccination plan | 70.7 | 91.0 |  |
| Make plan for campaign vaccination | 73.4 | 88.8 |  |
| Make annual immunization planning | 77.6 | 84.6 |  |
| Track the defaulters/drop-out rate | 54.1 | 79.8 |  |
| Identify the low proportion of DPT3 | 40.8 | 62.2 |  |
| Evaluate the data quality | 27.7 | 44.7 |  |
| Using NIIS data for other programs | 39.9 | 45.2 |  |
| Evaluate the performance of health facilities | 21.7 | 35.6 |  |
| Identify priority of supportive supervision | 19.1 | 30.9 |  |
| Make plan for vitamin A program | 27.1 | 26.6 |  |
| Identify the reasons and provide evidence for decision-making | 13.3 | 21.8 |  |

1. **Study population characteristics**

Immunization outcomes were assessed by comparing the NIIS data for a cohort of children pre- and post-intervention in the two provinces. After data cleaning, 81,485 children were included in the sample; their population characteristics are summarized in Table S8. In both provinces there were slightly more boys than girls. Nearly all children in Hanoi are Kinh and more than half live in rural areas. In Son La, there is a more distributed mix of ethnicities and nearly all children (more than 98 percent) live in rural areas. In Son La, nearly all children receive their vaccines from CHCs, whereas in Hanoi the majority receive their vaccines primarily from CHCs but there are also many children vaccinated primarily from FIFs. In Hanoi the percentage of children vaccinated primarily from FIFs increased from 28.8 percent in the pre-intervention cohort to 39.8 percent in the post-intervention cohort (p<.001).

Table S8. Study population characteristics.

| **Characteristics** | **Hanoi** | |  | **Son La** | |  |
| --- | --- | --- | --- | --- | --- | --- |
|  | **Pre**  **n=33,752** | **Post**  **n=35,611** | **p** | **Pre**  **n=6,233** | **Post**  **n=5,705** | **P** |
| **Child gender** |  |  |  |  |  |  |
| Girls | 47.1 | 47.1 | .95 | 47.7 | 46.4 | .16 |
| Boys | 52.9 | 52.9 |  | 52.3 | 53.6 |  |
| **Ethnicity** |  |  |  |  |  |  |
| Kinh | 99.5 | 99.4 | .002 | 23.4 | 23.3 | .002 |
| Thai | 0.1 | 0.1 |  | 45.2 | 43.1 |  |
| Other ethnicity | 0.4 | 0.5 |  | 31.4 | 33.7 |  |
| **Region** |  |  |  |  |  |  |
| Rural | 56.2 | 55.2 | .008 | 99.4 | 99.4 | .62 |
| Urban | 43.8 | 44.8 |  | 0.6 | 0.6 |  |
| **Vaccinated mostly from:** |  |  |  |  |  |  |
| Commune health center | 71.2 | 60.2 | <.001 | 98.7 | 99.2 | .006 |
| Fee-based immunization facility | 28.8 | 39.8 |  | 1.3 | 0.8 |  |

1. **Full immunization coverage results**

In Son La, full immunization coverage was significantly higher in the post-intervention cohort (88.3%) compared to the pre-intervention cohort (63.3%, p<.001) at 12 months. In Hanoi full immunization coverage was significantly lower in the post-intervention cohort (81.9%) compared to the pre-intervention cohort (87.8%, p<.001).

Table S9. Immunization coverage (at 12 months) for pre- and post-intervention cohorts by province.

| **Vaccine** | **Hanoi** | |  | **Son La** | |  |
| --- | --- | --- | --- | --- | --- | --- |
|  | **Pre**  **n = 33,752** | **Post**  **n = 35,611** | **p** | **Pre**  **n = 6,233** | **Post**  **n = 5,705** | **P** |
| BCG | 99.4 | 98.0 | <.001 | 97.8 | 98.9 | <.001 |
| Full polio | 95.2 | 93.6 | <.001 | 83.1 | 93.6 | <.001 |
| Penta 1 | 99.6 | 98.2 | <.001 | 94.2 | 98.3 | <.001 |
| Penta 2 | 98.9 | 97.0 | <.001 | 88.4 | 96.5 | <.001 |
| Penta 3 | 96.3 | 94.4 | <.001 | 75.5 | 93.4 | <.001 |
| Measles 1 | 93.4 | 84.8 | <.001 | 83.3 | 91.6 | <.001 |
| **Full immunization^1^** | 87.8 | 81.9 | <.001 | 63.3 | 88.3 | <.001 |

*^1^Full vaccination coverage, as defined by NEPI, is when a child has received his or her eight childhood vaccines: Bacillus Calmette-Guérin (BCG), 3 doses of hepatitis B, 3 doses of DPT, and 3 doses of Hib, 3 doses of polio (including at least one dose of IPV), and 1 dose of measles.*

**Hepatitis 1 included the hepatitis at birth.*

1. **Immunization dropout results**

Table S10. Drop-out rate before and after intervention by province.

| **Vaccine** | **Hanoi** | |  | **Son La** | |  |
| --- | --- | --- | --- | --- | --- | --- |
|  | **Pre**  **n=33,628** | **Post**  **n=34,970** | **P** | **Pre**  **n=5,940** | **Post**  **n=5,632** | **p** |
| Penta 1–Penta 3 | 2.3 | 3.5 | <.001 | 9.4 | 2.2 | <.001 |
| BCG–measles 1 | 1.7 | 7.7 | <.001 | 6.0 | 4.1 | .001 |

Table S11. Factors associated with drop-out of Penta1–Penta3 by province.

|  | **Hanoi** | | | **Son La** | | |
| --- | --- | --- | --- | --- | --- | --- |
|  | **n=68,646** | | | **n=11,605** | | |
|  | OR | p | 95% CI | OR | p | 95% CI |
| **Survey round** |  |  |  |  |  |  |
| Pre-intervention | Ref. |  |  | Ref. |  |  |
| Post-intervention | 1.6 | <.001 | [1.55,1.75] | .21 | <.001 | [0.18,0.26] |
| **Child gender** |  |  |  |  |  |  |
| Girls | Ref. |  |  | Ref. |  |  |
| Boys | 0.92 | .08 | [0.84,1.01] | .93 | .39 | [0.80,1.10] |
| **Ethnicity** |  |  |  |  |  |  |
| Kinh | 1.42 | .91 | [0.67,3.02] | 0.63^***^ | <.001 | [0.51,0.77] |
| Thai | 3.92 | .06 | [0.95,16.2] | 0.54^***^ | <.001 | [0.45,0.65] |
| Others ethnic minority | Ref. |  |  | Ref. |  |  |
| **Region** |  |  |  |  |  |  |
| Rural | Ref. |  |  |  |  |  |
| Urban | 3.7 | <.001 | [3.36,4.08] |  |  |  |
| **Vaccinated mostly from*** |  |  |  |  |  |  |
| Commune health center | Ref. |  |  | Ref. |  |  |
| Fee-based facility | 0.26 | <.001 | [0.23,0.28] | 0.25 | .051 | [0.06,1.00] |

**^¥^***We consider mostly as the child has received >4 vaccines out of 8 childhood vaccines recommended by NEPI.*

1. **Factors associated with on-time full immunization**

Table S12. Factors associated with on-time full immunization.

|  | **Hanoi** | | | **Son La** | | |
| --- | --- | --- | --- | --- | --- | --- |
|  | **n=69,301** | | | **n=11,918** | | |
|  | **OR** | **p** | **95% CI** | **OR** | **P** | **95% CI** |
| **Survey round** |  |  |  |  |  |  |
| Pre-intervention | Ref. |  |  | Ref. |  |  |
| Post-intervention | 0.61 | <.001 | [0.58,0.63] | 4.47 | <.001 | [4.06,4.92] |
| **Province** |  |  |  |  |  |  |
| Hanoi |  |  |  |  |  |  |
| Son La |  |  |  |  |  |  |
| **Child gender** |  |  |  |  |  |  |
| Girls | Ref. |  |  | Ref. |  |  |
| Boys | 1.01 | .667 | [0.97,1.05] | 1.01 | .77 | [0.93,1.11] |
| **Ethnicity** |  |  |  |  |  |  |
| Kinh | 0.85 | .378 | [0.60,1.21] | 1.26 | <.001 | [1.12,1.42] |
| Thai | 0.46 | .087 | [0.20,1.12] | 1.66 | <.001 | [1.50,1.84] |
| Others ethnic minority | Ref. |  |  | Ref. |  |  |
| **Region** |  |  |  |  |  |  |
| Rural | Ref. |  |  |  |  |  |
| Urban | 0.21 | <.001 | [0.20,0.22] |  |  |  |
| **Vaccinated mostly from*** |  |  |  |  |  |  |
| Commune health center | Ref. |  |  | Ref. |  |  |
| Fee-based facility | 1.72 | <.001 | [1.64,1.80] | 1.27 | .28 | [0.83,1.96] |

**^¥^***We consider mostly as the child has received >4 vaccines out of 8 childhood vaccines recommended by NEPI.*
